# Proteomic responses of oceanic *Synechococcus* WH8102 to phosphate and zinc scarcity and cadmium additions

**DOI:** 10.3389/fmicb.2013.00387

**Published:** 2013-12-17

**Authors:** Alysia D. Cox, Mak A. Saito

**Affiliations:** ^1^MIT/WHOI Joint Program in Chemical Oceanography, Massachusetts Institute of Technology and Woods Hole Oceanographic InstitutionWoods Hole and Cambridge, MA, USA; ^2^Marine Chemistry and Geochemistry Department, Woods Hole Oceanographic InstitutionWoods Hole, MA, USA

**Keywords:** cyanobacteria, *Synechococcus*, zinc, cadmium, phosphate stress, proteome, metallothionein, alkaline phosphatase

## Abstract

*Synechococcus* sp. WH 8102 is a motile marine cyanobacterium isolated originally from the Sargasso Sea. To test the response of this organism to cadmium (Cd), generally considered a toxin, cultures were grown in a matrix of high and low zinc (Zn) and phosphate (PO_4_^3−^) and were then exposed to an addition of 4.4 pM free Cd^2+^ at mid-log phase and harvested after 24 h. Whereas Zn and PO_4_^3−^ had little effect on overall growth rates, in the final 24 h of the experiment three growth effects were noticed: (i) low PO_4_^3−^ treatments showed increased growth rates relative to high PO_4_^3−^ treatments, (ii) the Zn/high PO_4_^3−^ treatment appeared to enter stationary phase, and (iii) Cd increased growth rates further in both the low PO_4_^3−^ and Zn treatments. Global proteomic analysis revealed that: (i) Zn appeared to be critical to the PO_4_^3−^ response in this organism, (ii) bacterial metallothionein (SmtA) appears correlated with PO_4_^3−^ stress-associated proteins, (iii) Cd has the greatest influence on the proteome at low PO_4_^3−^ and Zn, (iv) Zn buffered the effects of Cd, and (v) in the presence of both replete PO_4_^3−^ and added Cd the proteome showed little response to the presence of Zn. Similar trends in alkaline phosphate (ALP) and SmtA suggest the possibility of a Zn supply system to provide Zn to ALP that involves SmtA. In addition, proteome results were consistent with a previous transcriptome study of PO_4_^3−^ stress (with replete Zn) in this organism, including the greater relative abundance of ALP (PhoA), ABC phosphate binding protein (PstS) and other proteins. Yet with no Zn in this proteome experiment the PO_4_^3−^ response was quite different including the greater relative abundance of five hypothetical proteins with no increase in PhoA or PstS, suggesting that Zn nutritional levels are connected to the PO_4_^3−^ response in this cyanobacterium. Alternate ALP PhoX (Ca) was found to be a low abundance protein, suggesting that PhoA (Zn, Mg) may be more environmentally relevant than PhoX.

## Introduction

The marine Cyanobacteria *Synechococcus* and *Prochlorococcus* contribute between 32 and 80% of the total primary productivity in oligotrophic oceans (Goericke and Welschmeyer, 1993; Li, 1995; Liu et al., 1997; Veldhuis et al., 1997; Rocap et al., 2002) and about 50% of the fixed carbon in some oceanic regions (Zwirglmaier et al., 2007). In addition, the Marine Cluster-A group (MC-A or *Synechococcus* subcluster 5.1) is thought to be the dominant *Synechococcus* group within the euphotic zone of open ocean and coastal waters (Fuller et al., 2003). *Synechococcus* WH8102 is a well-studied Sargasso Sea isolate from the MC-A group with an available genome sequence (Waterbury et al., 1986; Scanlan, 2003; Palenik et al., 2003). Previous culture studies examining the influences of metals on this organism showed that at low zinc (Zn) concentrations increased cadmium (Cd) concentrations inhibited growth, whereas this was not observed at higher Zn concentrations (Saito et al., 2003).

Cd and Zn have nutrient-like distributions in the ocean, meaning they are depleted in surface waters and increase with depth, implying that Cd and Zn are taken up by microorganisms in the surface water and remineralized at depth (Boyle et al., 1976; Bruland, 1980). Dissolved total Zn may reach concentrations up to ~9 nM at depth, whereas Cd may reach up to ~1 nM (Bruland, 1980, 1992). Notably, this excess of dissolved Zn over Cd is typical of deepwater ocean environments, however, this difference can decrease in surface waters as Zn is depleted (Sunda and Huntsman, 2000; Saito et al., 2010). Zn is important to the proper functionality of many enzymes and is thought to be an essential metal in living organisms, whereas Cd is only known to be used in some carbonic anhydrases of diatoms (Morel et al., 1994; Lee et al., 1995; Lane and Morel, 2000; Lane et al., 2005; Park et al., 2007; Xu et al., 2008). As a result, these metals may have different roles in different environments and organisms. Zn is a nutrient in the open ocean and has been suggested to influence phytoplankton diversity in the Ross Sea (Saito et al., 2010). In cyanobacteria, the Zn requirements appear to be very low, consistent with the idea that cyanobacteria may have evolved in a sulfidic or ferruginous ancient ocean when Zn was strongly complexed and of low bioavailability (Saito et al., 2003; Robbins et al., 2013). A coastal cyanobacterium, *Synechococcus bacillaris* showed no requirement for Zn (Sunda and Huntsman, 1995). In addition, low Zn abundances were shown to have little to no effect on the growth rates of the related marine cyanobacterium *Prochlorococcus marinus* strain MED4 (Saito et al., 2002). Notably these Zn limitation studies were conducted with replete inorganic phosphate and no added organic phosphate. Perhaps because of the low Zn requirement and trace metal culturing techniques required to perform such investigations, there are few studies of intracellular Zn homeostasis mechanisms in marine cyanobacteria (Blindauer, 2008). In terms of Cd, it has been noticed that the dissolved Cd:PO_4_^3−^ ratios are lower in the surface waters of iron-limited regions, implying preferential removal of Cd relative to PO_4_^3−^ in iron-limited waters, perhaps due to Cd transport through ferrous iron transporters or prior depletion of Zn (Cullen, 2006; Lane et al., 2009; Saito et al., 2010). As a result, the potential interactions between Cd and Zn in the ocean range from biochemical substitution in diatoms (Morel et al., 1994; Lee et al., 1995; Lane and Morel, 2000; Lane et al., 2005) to antagonistic effects in cyanobacteria.

Cd has been suspected to interact with Zn in organisms for over half a century. Early mentions of this concept stated that in certain fungi Cd cannot physiologically replace Zn (Goldschmidt, 1954), and recent studies have shown that Cd can restore growth in Zn-limited marine diatoms (Price and Morel, 1990; Lee and Morel, 1995; Sunda and Huntsman, 2000). In marine cyanobacteria the intracellular destination of Cd is likely metallothionein, but other possibilities exist such as low molecular weight thiols, polyphosphates or metalloenzymes like carbonic anhydrase (Cox, 2011). A connection of Zn and perhaps Cd to phosphate exists due to the Zn metalloenzyme alkaline phosphatase that is used by marine microbes in the acquisition of organic phosphate. Bacterial cells have evolved complicated mechanisms to ensure that metalloproteins contain the correct metal, but the processes are not perfect and elucidating these mechanisms may require a systems-based approach (Waldron and Robinson, 2009). In this study, by adding Cd to a Zn-scarce environment, we are exposing cells to a metal to which they are unaccustomed in order to discern cellular processing of these specific metals by observing the protein system response.

Phosphorus is an essential nutrient, utilized in the cell as part of large biomolecules (DNA, RNA, phospholipids), for chemical energy transfer (adenine triphosphate, ATP), in cellular signaling networks, and in reversible chemical modification of proteins. It is typically found at low micromolar to nanomolar concentrations in the ocean and is limiting in some regions. It composes some 2–4% dry weight of cells (Karl, 2000). Scarcity of both phosphorus and Zn could result in biochemically dependent colimitation, in which the uptake of organic phosphorus, is dependent upon Zn adequate nutrition due to its role in alkaline phosphatase (Saito et al., 2008). It has been hypothesized that Zn and phosphorus colimitation could occur in oligotrophic regions such as the Sargasso Sea, based on laboratory experiments with the coccolithophore *Emiliania huxleyi* (Shaked et al., 2006).

In this manuscript, the physiological and proteomic responses of the open ocean *Synechococcus* WH8102 to acute Cd exposure under varying chronic Zn and PO_4_^3−^ concentrations were examined to (1) probe Zn use in the organism and how it deals with an interfering metal (Cd), (2) investigate potential ecological and biogeochemical significance of Cd and Zn interactions, (3) investigate the effects of Cd on phosphate stress proteins and (4) investigate the influence of Zn on phosphate stress. Given that Zn is found in excess of Cd in oceanic habitats, reversing this relationship with short-term Cd enrichments provides opportunities to probe metal homeostasis in cyanobacteria. The proteome response in high and low concentrations of phosphate showed distinct responses in alkaline phosphatase and metallothionein with Cd or Zn suggesting that the regulatory system responds to the two metals differently (Zn is currently thought to be vital at low phosphate whereas Cd is not). Alkaline phosphate and metallothionein show similar responses suggesting a Zn-handling mechanism in which metallothionein supplies alkaline phosphatase with Zn may exist.

## Methods

### Culturing

**Figure 1:**
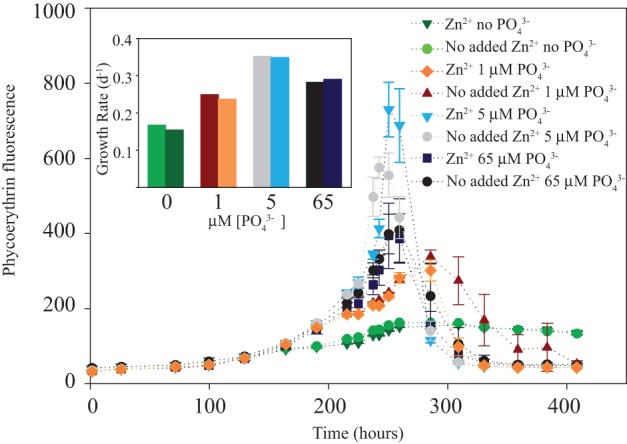
**Phycoerythrin fluorescence vs. time, chronic PO_4_^3−^ limitation reconnaissance study**. Error bars are one standard deviation of triplicate 28 mL tubes. Note that no PO_4_^3−^ added treatments, both with and without Zn appear to have a stationary phase. 1 μM PO_4_^3−^ treatments appear to have a brief stationary phase and then enter death phase, the Zn dying faster than the no Zn. The 5 μM PO_4_^3−^ treatments fluoresced to a greater maximum than the 65 μM PO_4_^3−^.

Axenic cultures of *Synechococcus* sp. WH8102 were obtained from J. Waterbury and F. Valois (Woods Hole Oceanographic Institution) and maintained in a PRO-TM media [modified from (Saito et al., 2002)] made with 75% oligotrophic seawater obtained from the oligotrophic South Atlantic ocean and prepared by microwave sterilization and the addition of chelexed and sterile filtered nutrients [1.1 mM NO^−^_3_ and 65 μM PO_4_^3−^] and ethylenediaminetetraacetic acid (EDTA)-complexed metals (22.2 μM EDTA, 171 nM MnCl_2_, 5.7 nM Na_2_MoO_4_, 19 nM Na_2_SeO_3_, 2.22 μM FeCl_3_, 19 nM CoCl_2_, 19 nM NiCl_2_). The scarce Zn^2+^ condition had no Zn added whereas replete had Zn added to a total concentration of 10 nM, with the free concentrations estimated to be tens of picomolar Zn^2+^ (Saito et al., 2003). Low PO_4_^3−^ cultures had 1 μM PO_4_^3−^ added, whereas high had 65 μM PO_4_^3−^. The ideal low concentration of PO_4_^3−^ for this experiment was determined in a reconnaissance study with concentrations ranging from no added PO_4_^3−^ to 65 μM PO_4_^3−^ added (Figure 1). Acute Cd treatments had Cd added to a total concentration of 10 nM CdCl_2_, with the free concentrations estimated to be 4.4 pM Cd^2+^ using EDTA stability constant data from Smith and Martell (1993). The ratio of Cd^2+^: Cd_TOT_ was calculated to be 1:2267. This ratio in a PRO-TM media with 11.7 μM EDTA (Saito et al., 2002) was calculated to be 1:1216 (Saito et al., 2003) and 1:6026 in a media with 100 μM EDTA (Sunda and Hunstman, 1998). The ratio of Cd^2+^ to the total of major inorganic species in a PRO-TM media with 11.7 μM EDTA (Saito et al., 2002) was calculated to be 1:36 (Saito et al., 2003). The blank of the medium was not determined. Previous researchers doing similar trace metal culture studies have assumed background metal concentrations of 100 pM for cobalt (Sunda and Huntsman, 1995; Sunda and Hunstman, 1998; Saito et al., 2002), 900 pM for zinc (Sunda and Huntsman, 1995; Sunda and Hunstman, 1998) and 100 pM for cadmium (Sunda and Hunstman, 1998).

**Figure 2:**
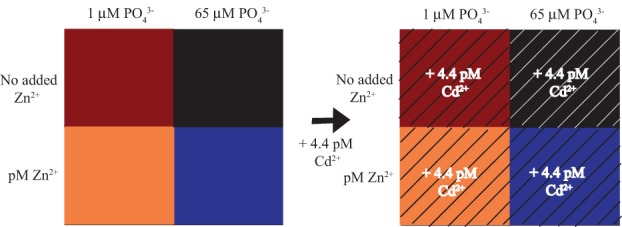
**Experimental Design**. Four experimental treatments with variable Zn and PO_4_^3−^ concentrations were grown to mid-log phase, split evenly and 4.4 pM Cd^2+^ added acutely to one of the splits of each treatment.

**Figure 3:**
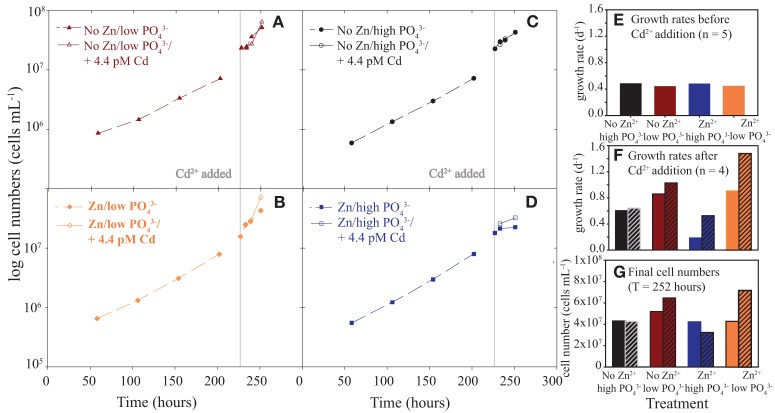
**Cell numbers vs. time, growth rates, and final cell numbers. (A)** no Zn/low PO_4_^3−^ with and without short-term Cd addition, **(B)** Zn/low PO_4_^3−^ with and without short-term Cd addition, **(C)** no Zn/high PO_4_^3−^ with and without short-term Cd addition, **(D)** Zn/high PO_4_^3−^ with and without short-term Cd addition, **(E)** growth rates, **(F)** growth rates in the 24 h after Cd addition until harvest and **(G)** final cell numbers at harvest. Vertical lines mark time of Cd addition. Note that final cell numbers are higher in low than high phosphate. *n*, number of timepoints.

Cultures were grown in either 28 mL polycarbonate tubes or 500 mL polycarbonate bottles under 30 μE m^−2^s^−1^ continuous white light. At mid-log phase, the four 500 mL cultures were split and 4.4 pM Cd^2+^ added to one of each treatment (hereafter Cd addition). The 8 resulting cultures were harvested 24 h later (Figure 2). Culture growth was monitored by a combination of chlorophyll *a* and phycoerythrin fluorescence and cell counting by microscopy. All plasticware was soaked for two days in a detergent, then two weeks in 10% HCl (Fisher, trace metal grade), rinsed with pH 2 HCl and then microwave sterilized. Growth rates were calculated from the slope of the natural log of *in vivo* relative chlorophyll *a* fluorescence (*n* = 5 timepoints, Figure 3). For protein samples, approximately 200 mL of culture were harvested by centrifugation in a Beckman J2-21M centrifuge at 18,566 g for 30 min at 4°C, decanted, transferred into a microtube and centrifuged again at 14,000 g for 15 min at room temperature, decanted, and frozen at −80°C.

### Protein extraction

Protein was extracted from the digestion of frozen whole cell pellets. Sample tubes were kept on ice throughout the extraction process, unless otherwise noted. Cell pellets were resuspended in 500 μL of ice-cold 100 mM ammonium bicarbonate buffer solution, pH 8.0 (AMBIC). Samples were sonicated on ice using a Branson sonifier 450 for 4 min at 70% duty with an output level of 3, allowed a 5 min pause, then sonicated for another 4 min. Samples were then centrifuged at 4°C at 14,000 g for 35 min. 200 μL of supernatant were precipitated overnight with 800 μL of −20°C acetone.

Acetone-precipitated samples were centrifuged at 4°C at 14,000 g for 30 min and decanted. One hundred μL of freshly made 7.5 M urea in AMBIC and 25 μL of AMBIC were added to the acetone-precipitated pellet. Samples were incubated for approximately 15 min at room temperature with periodic vortexing then resuspended by incubation for 5 min at 95°C. A 100 μL aliquot was removed and 5 μL of 200 mM dithiothreitol (DTT) in AMBIC were added and incubated for 1 h at 56°C, shaken at 400 rpm. The samples were vortexed and centrifuged at 14,000 g for 2 min. Twenty μL of 200 mM iodacetamide in AMBIC were added and incubated for 1 h at room temperature in the dark, shaken at 400 rpm. 20 μL of 200 mM DTT in AMBIC were added, mixed, centrifuged for 2 min as above, and incubated for 1 h at room temperature, shaken at 400 rpm. After incubation, samples were centrifuged for 2 min as above. Total protein yield was assayed using the Biorad DC Protein Assay. Trypsin (Promega) was reconstituted in 500 μL of 50 mM acetic acid and added in a trypsin to protein ratio of 1:50. The samples were mixed, vortexed, centrifuged for 2 min as above, and incubated for approximately 16 h at 37°C, shaken at 400 rpm.

After trypsin digestion, samples were vortexed, centrifuged for 2 min, and 20 μL of LC-MS grade glacial acetic acid added. Samples were evaporated by speed vacuum for approximately 3 h to a final volume of approximately 600 μL. The samples were centrifuged at 14,000 g for 30 minutes and the supernatants collected. Four micrograms of protein were injected for LC-MS.

### Liquid chromatography-mass spectrometry (LC-MS)

The digests were analyzed by LC-MS using a Microhm Paradigm MS4 HPLC system with reverse phase chromatography, Thermo LTQ ion trap mass spectrometer and Microhm ADVANCE source [2 μL/min flow rate; reversed phase Magic C18 AQ column, 0.2 × 150 mm, 3μm particle size, 200Å poresize; 345 min runs; hyperbolic gradient of water to acetonitrile (each containing 0.1% formic acid)]. Each digest was injected three times for a total of 24 mass spectrometry runs; only two runs from each treatment were analyzed. Mass spectra were processed by SEQUEST and PeptideProphet with a fragment tolerance of 1.0 Da (monoisotopic), parent tolerance of 2.0 Da (monoisotopic, fixed modification of +57 on C (carbamidomethyl), variable modification of +16 on M (oxidation) and a maximum of 2 missed trypsin cleavages using a database including reversed proteins and common contaminants.

Spectral counts of 16 files were compiled in Scaffold 3 Proteome Software with a peptide false discovery rate of 1.9%, minimum peptide and protein tolerances of 95 and 99%, respectively, with a minimum of 2 peptides (Peng et al., 2003; Zhang et al., 2006). A spectral count is the number of times a particular peptide from a protein is sampled during an MS/MS experiment and the normalized spectral count is indicative of protein relative abundance. Protein functions were assigned manually using the Kyoto Encyclopedia of Genes and Genomes (KEGG) unless otherwise noted.

### Pairwise analyses and fisher's exact test

Proteins were considered differentially abundant in the pairwise analyses if the average spectral count value of one of the pairs was equal to or greater than five and the pair of proteins different by two-fold or more. Use of Fisher's Exact Test (Zhang et al., 2006) confirms that most proteins are different in abundance using these stringencies, excepting a few proteins with five spectral counts. The two-fold or more differentially abundant proteins with low spectral counts remain in the tables, but are considered tenuous in analysis. The results of Fisher's Exact Test also conclude that more proteins are statistically different in abundance than the greater than or equal to two-fold analysis alone. This is because a smaller fold difference in a greater value is statistically different, thus proteins with higher spectral counts that are different by less than two-fold are differentially abundant.

## Results

### Physiological data

Growth limiting PO_4_^3−^ concentrations for *Synechococcus* WH8102 were determined in a reconnaissance experiment to occur at no added and 1 μM PO_4_^3−^ (Figure 1). No added PO_4_^3−^ treatments had very low biomass and so 1 μM was chosen for the low PO_4_^3−^ treatment and 65 μM for the high PO_4_^3−^ in subsequent proteomic experiments. This slightly contrasts the transcriptome study of Tetu et al. (2009), where *Synechococcus* WH8102 was PO_4_^3−^ stressed at 5 μM.

*Synechococcus* WH8102 was grown in a matrix of Zn (Zn or no Zn hereafter, no Zn treatment also referred to as “scarce”) and PO_4_^3−^ conditions to examine the potential interactions (Figure 2). In late log phase, cultures were split and an environmentally relevant amount of Cd was added to one split (4.4 pM Cd^2+^, 10 nM Cd_TOT_) to test the Cd response. Responses were monitored by phycoerythrin and chlorophyll *a in vivo* fluorescence and cell counts every 48 h during the 11-day experiment and four times in the last 24 h for the short-term Cd addition experiment (cell abundances in Figure 3, fluorescence data in Cox, 2011). These growth curves revealed four main observations: First, growth rates of the Zn/PO_4_^3−^ matrix prior to Cd addition were similar, the low PO_4_^3−^ treatments with slightly lower growth rates (Figure 3E). Growth rates were calculated using cell abundances (Figures 3A–D), rather then fluorescence (Figure 1). Second, the Zn/high PO_4_^3−^ treatment appeared to enter a stable stationary phase relative to other treatments (Figures 3D,F). Third, low PO_4_^3−^ treatments showed increased instantaneous growth rates relative to high PO_4_^3−^ during the final 24 h of the experiment (Figure 3F). Physical perturbation of the cultures by splitting them may have caused a different response in the low and high PO_4_^3−^ treatments. Last, Cd addition increased instantaneous growth rates even further above the low PO_4_^3−^ and Zn treatments (Figure 3F). Final cell numbers at harvest for protein biomass were similar for most treatments, but showed slightly elevated cell numbers for two treatments, no Zn/low PO_4_^3−^/short-term Cd and Zn/low PO_4_^3−^/short-term Cd (Figure 3G).

### Global proteomic data

Analysis resulted in the identification of 483 proteins and 3947 unique peptides from 62,264 mass spectra over 16 LC-MS injections, 8 treatments injected in duplicate (Data Sheet 1). Using the peptide prophet algorithm in Scaffold 3, 95% peptide minimum confidence level, 99.9% protein minimum confidence level and a minimum of 2 peptides per protein identification resulted in a 1.9% peptide false discovery rate (Peng et al., 2003; Zhang et al., 2006) (Data Sheet 1). This experiment identified 24% of the 2519 possible proteins present in the genome of WH8102. Using the same conditions mentioned above but with a more stringent minimum of 3 peptides per protein identification resulted in 420 protein identifications with a 0.9% peptide false discovery rate. Seventy-one proteins showed differences in protein abundances in at least two treatments using a minimum difference of 7 spectral counts and a threshold of 7 spectral counts, based on technical replicates of each of the 8 treatments.

**Figure 4:**
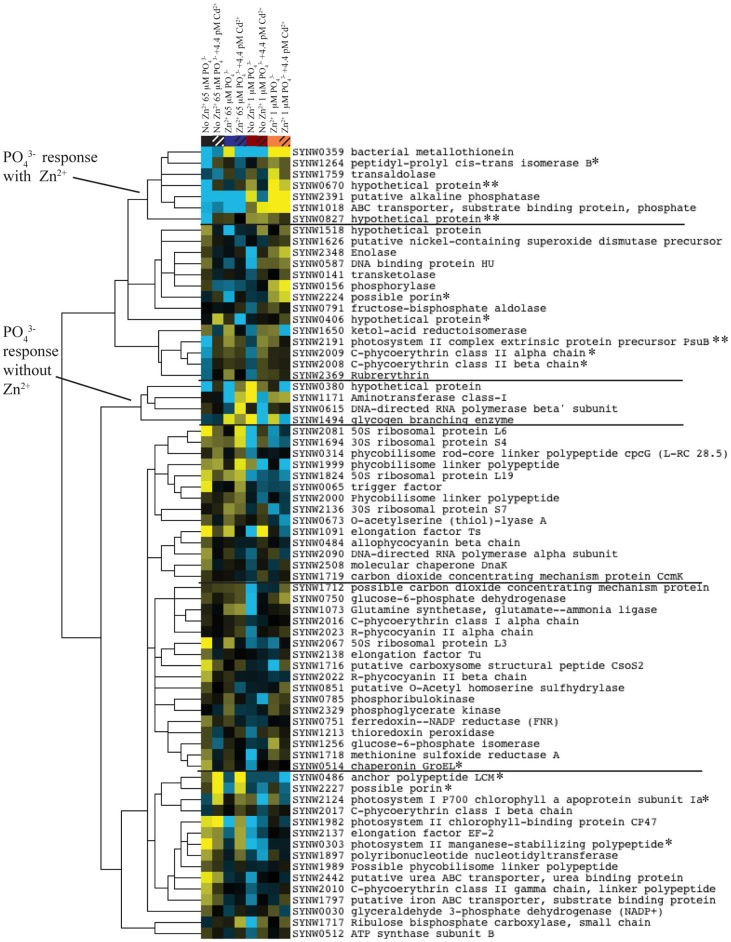
**Cluster analysis of relative protein abundances**. no Zn/65 μM PO_4_^3−^, Zn/65 μM PO_4_^3−^, no Zn/1 μM PO_4_^3−^, Zn/1 μM PO_4_^3−^ and these four chronic treatments with short term 4.4 pM Cd^2+^ added. The four low PO_4_^3−^ treatments are on the right and replete, and high PO_4_^3−^ on the left. There are 71 proteins. Protein relative abundances are averages of duplicates, have at least 7 counts, and are different by a value of 7. Data are log transformed, centered, and clustered by Kendall's Tau, centroid linkage. Yellow, more abundant; Blue, less abundant; ^*^, statistically different by Fisher's Exact Test between the no Zn/high PO_4_^3−^ and the no Zn/high PO_4_^3−^/short-term Cd; ^**^, differentially abundant by two-fold or greater and statistically different by Fisher's Exact Test between the no Zn/high PO_4_^3−^ and the no Zn/high PO_4_^3−^/short-term Cd.

**Figure 5:**
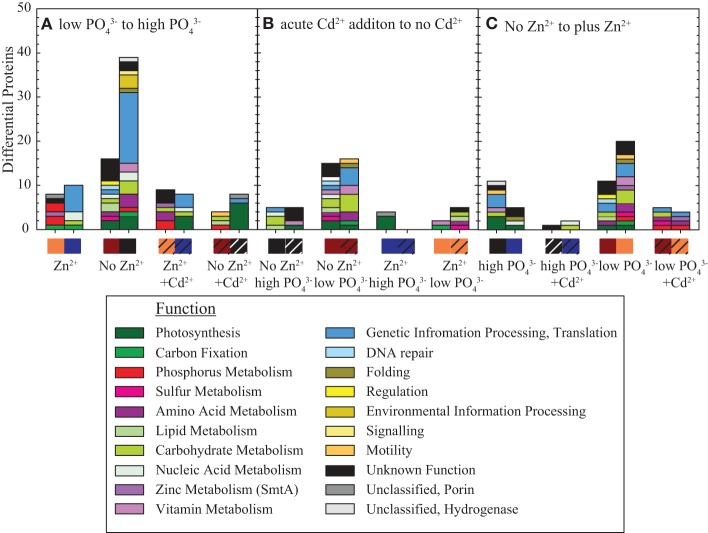
**Number of proteins two-fold or more abundant in pairwise comparisons, with other matix treatments equivalent (color bar on x-axis corresponds with Figure 2 design), with protein bars colored by KEGG function. (A)** Proteins more abundant with scarce PO_4_^3−^ relative to replete conditions and vice versa (right and left bars, respectively). **(B)** Proteins more abundant with short-term Cd addition relative to no Cd added and vice versa (right and left bars, respectively). **(C)** Proteins more abundant with scarce Zn relative to replete conditions and vice versa (left and right bars, respectively). Comparison of **(A–C)** shows that the combination of Zn and PO_4_^3−^ scarcity causes the greatest number of proteins to be differentially abundant. **(B)** shows that the presence of Zn causes less change in number of proteins when Cd is added. Functions and abundances for each protein are in Tables 1–3 and Supplementary Tables 1A,B, and D–J.

Both cluster analysis (Figure 4; Datasheet 1; Eisen et al., 1998) and pairwise comparisons between experimental treatments foremost reveal PO_4_^3−^ stress effects, and next Cd and Zn effects (Figure 5; Tables 1–3; Supplementary Tables 1A–K). Each column represents a number of proteins that are at least two-fold different in abundance when a single condition is varied and the protein bars are coded by function (Figure 5). In these pairwise proteome comparisons, three observations can be made. First, the no Zn/low PO_4_^3−^ treatment had the greatest number of proteins that were two-fold different in abundance among all pairwise comparisons (55 in Figure 5A, 32 in Figure 5B and 31 in Figure 5C). In contrast, the no Zn/high PO_4_^3−^ treatment had 55 different proteins in Figure 5A [same number because directly compared to the low PO_4_^3−^], 10 in Figure 5B and 16 in Figure 5C. Second, Cd addition caused a greater change in the number of two-fold different proteins when Zn was absent (Figure 5B). The presence of Zn caused a smaller change in the total number of proteins of two-fold difference when Cd was added [42 with no Zn, both high and low PO_4_^3−^ to 11 proteins in the presence of Zn, both high and low PO_4_^3−^; Figure 5B]. Third, short-term Cd addition under both low and high PO_4_^3−^ conditions had fewer proteins of two-fold difference than in the presence or absence of Zn, suggestive of possible Cd alleviation of Zn deprivation (Figure 5C). These observations imply the Zn deprivation combined with PO_4_^3−^ stress causes the greatest number of proteins to be differentially abundant, the proteome responds more with Cd addition without added Zn in the media, and Zn alleviates Cd addition effects at both high and low PO_4_^3−^.

**Table 1:** **Relative protein abundances between low and high phosphate treatments for proteins two-fold or greater differentially abundant (1 μM PO_4_^3−^ and 65 μM PO_4_^3−^, replete Zn for both)**.

**SYNW ID**	**KEGG function**	**Protein**	**1 μM PO_4_^3−^**	**65 μM PO_4_^3−^**	**Low/high fold change**
2391^*^	u,p	Putative alkaline phosphatase	8.1 ± 0.8	1.0 ± 0.0	+8.1
1018^*^	abc,p	ABC transporter, substrate binding protein, phosphate (PstS)	76.9 ± 1.3	19.2 ± 2.4	+4.0
1661	ukn	Hypothetical protein	5.2 ± 2.1	1.4 ± 0.7	+3.7
0953^*^	mo	Cell surface protein required for swimming motility (SwmB)	5.2 ± 0.6	1.4 ± 0.7	+3.7
0359	u,zn	Bacterial metallothionein (SmtA)	7.1 ± 3.2	3.3 ± 0.6	+2.2
0085^*^	mo	Cell surface protein required for swimming motility (SwmA)	9.0 ± 0.8	4.2 ± 0.6	+2.1
0799^*^	m,e,c	Glyceraldehyde−3−phosphate dehydrogenase (Gap3)	2.4 ± 0.6	0.5 ± 0.7	+4.7
2224^*^	u,om	Possible porin (Som)	61.2 ± 1.7	29.4 ± 2.6	+2.1
1773	m,nu,pu,a	Adenylosuccinate synthetase (PurA, AdeK)	1.4 ± 0.7	7.5 ± 0.1	−5.2
0814	m,nu, pu	Adenine phosphoribosyltransferase	1.0 ± 0.0	5.1 ± 0.8	−5.1
2500	m,cb,tca, e,c	Aconitate hydratase (AcnB)	2.8 ± 0.0	7.5 ± 0.1	−2.6
2069	gi,t	50S ribosomal protein L23 (Rpl23,RplW)	3.8 ± 1.3	8.9 ± 0.5	−2.3
2068	gi,t	50S ribosomal protein L4 (Rpl4,RplD)	4.3 ± 2.1	9.3 ± 0.2	−2.2
2079	gi,t	50S ribosomal protein L5 (Rpl5,RplE)	5.2 ± 1.9	10.7 ± 0.4	−2.1
1716	c	Putative carboxysome structural peptide (CsoS2)	6.6 ± 0.1	13.1 ± 1.6	−2.0
2136	gi,t	30S ribosomal protein S7 (Rps7,RpsG)	8.1 ± 0.5	15.9 ± 1.0	−2.0
2083	gi,t	30S ribosomal protein S5 (Rps5,RpsE)	5.2 ± 0.8	10.3 ± 0.2	−2.0
2082^#^	gi,t	50S ribosomal protein L18 (Rpl18,RplR)	3.3 ± 0.6	6.5 ± 2.5	−2.0

*Units are spectral counts*.

* Corresponding transcript identified in Tetu et al. (2009) as strongly upregulated under early P-stress [5 μM PO_4_^3−^];

# *Corresponding transcript identified in Tetu et al. (2009) as strongly downregulated under early P-stress [5 μM PO_4_^3−^]; u, unclassified; p, phosphorus metabolism; abc, ABC transporter; ukn, unknown; mo, motility; zn, zinc metabolism; m, metabolism; e, energy metabolism; c, carbon fixation; om, outer membrane protein; nu, nucleic acid metabolism; pu, purine metabolism; a, amino acid metabolism; cb, carbohydrate metabolism; tca, citrate cycle; gi, genetic information processing; t, translation*.

**Figure 6:**
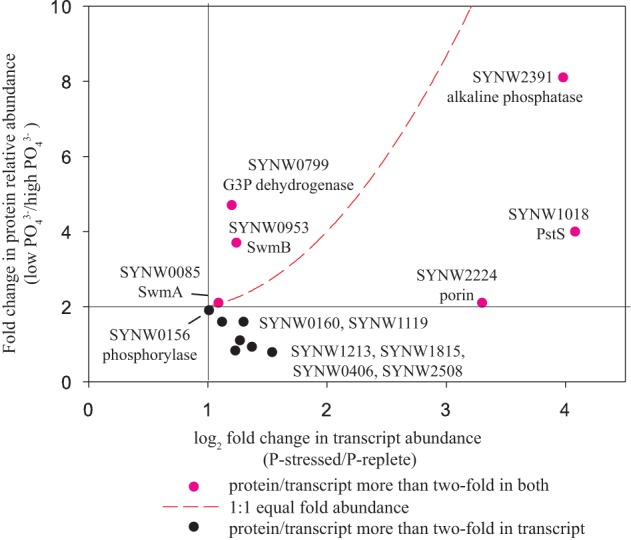
**Fold change in protein relative abundance (this experiment) as ratio of low phosphate to high phosphate vs. log_2_ fold change in gene relative abundance (Tetu et al., 2009) as ratio of P-stressed to P-replete**. Pink dots represent proteins/transcripts more than two-fold abundant in both protein and transcript data. Black dots represent proteins/transcripts more than two-fold abundant in transcript data. Red dashed line indicates a 1:1 equal fold abundance. SYNW0160 conserved hypothetical protein; SYNW1119 6-phosphogluconate dehydrogenase; SYNW1213 thioredoxin peroxidase; SYNW1815 ABC transporter, substrate binding protein, phosphate; SYNW0406 hypothetical protein; SYNW2508 molecular chaperone DnaK2, heat shock protein hsp 70-2. See Tables 1, 2.

**Figure 7:**
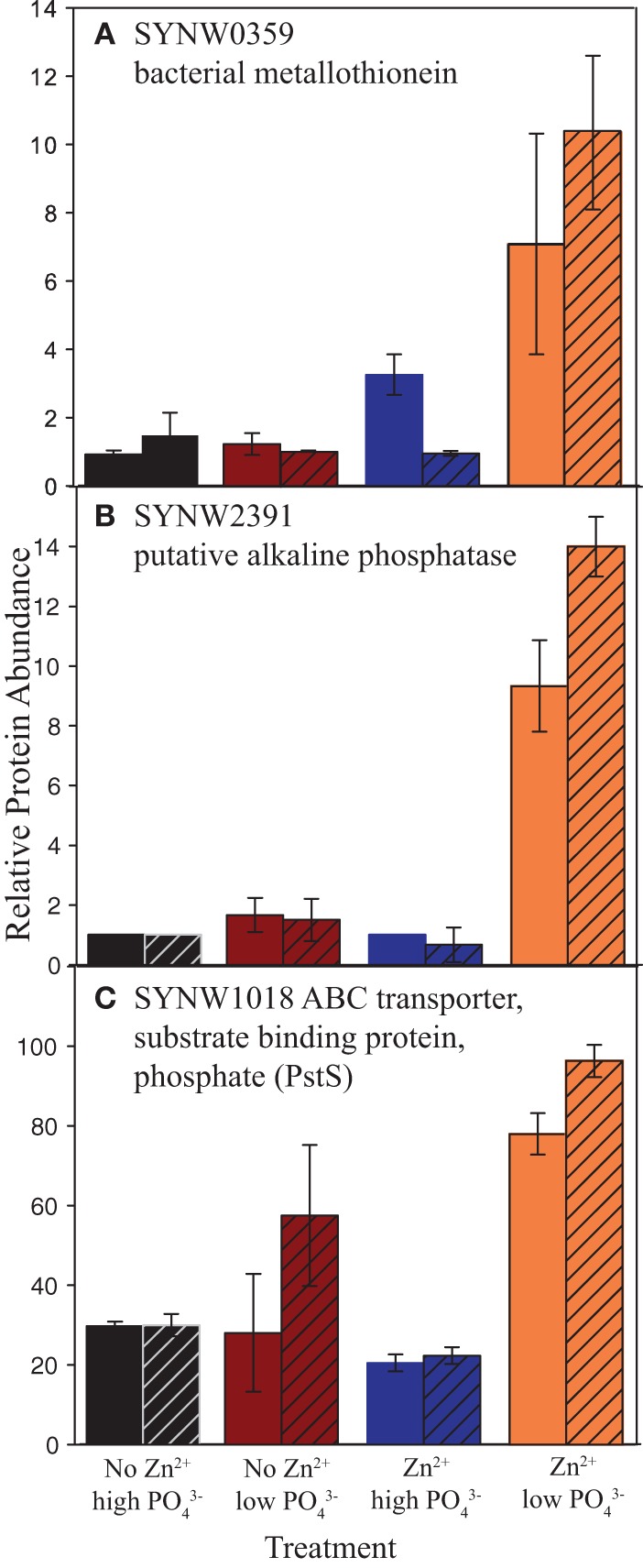
**Relative protein abundances of SYNW0359 bacterial metallothionein, SYNW2391 putative alkaline phosphatase, and SYNW1018 ABC transporter, substrate binding protein, phosphate (PstS)**. Hatched bars were subjected to short-term Cd additions. Error bars are the standard deviation of duplicate injections. Note greater relative abundances of the alkaline phosphatase and PstS in the 1 μM PO_4_^3−^ compared to the 65 μM PO_4_^3−^ treatments. Note the greater relative abundances of the alkaline phosphatase in the 1 μM PO_4_^3−^ treatments with Zn compared to no Zn.

The proteome response to high and low PO_4_^3−^ (in the Zn treatments) was similar to the transcriptome results by Tetu et al. (2009), which used the same *Synechococcus* strain (Figure 6). Eighteen total proteins were two-fold or more differentially abundant (with a spectral count threshold of at least five) between 1 and 65 μM PO_4_^3−^ treatments, (Table 1). Eight proteins were more abundant in the 1 μM PO_4_^3−^ treatment, including 6 proteins found to be similarly differentially expressed in the transcriptome (Figure 6, starred in Table 1). These 6 proteins include a putative alkaline phosphatase and a phosphate binding protein component of an ABC transporter (PstS). Also of note is a bacterial metallothionein that was not observed in the microarray experiment. The metallothionein, alkaline phosphatase, and phosphate transporter also show higher relative abundances at low PO_4_^3−^ with increased Zn abundance (Figure 7). Six of the ten proteins more abundant in the 65 μM PO_4_^3−^ treatments were ribosomal proteins and one of these was downregulated as a transcript (50S ribosomal protein L18, Table 1).

In addition to PO_4_^3−^ effects alone, we examined the PO_4_^3−^ response with and without added Zn. Table 2 lists the 55 proteins with differential responses at low PO_4_^3−^. Sixteen proteins were more abundant in the low PO_4_^3−^ treatment, including five hypothetical proteins and two proteins involved in photosynthesis. Under low Zn no proteins showed abundance trends similar to gene expression in the microarray experiment. Note that metallothionein, alkaline phosphatase and the ABC transporter, phosphate substrate binding protein were less abundant in the low PO_4_^3−^ without Zn than with Zn (Figure 7).

**Table 2:** **Relative protein abundances between low and high phosphate treatments for proteins two-fold or greater differentially abundant (1 μM PO_4_^3−^ and 65 μM PO_4_^3−^, scarce Zn for both)**.

**SYNW ID**	**KEGG function**	**Protein**	**1 μM PO_4_^3−^**	**65 μM PO_4_^3−^**	**Low/high fold change**
0380	ukn	Hypothetical protein	13.5 ± 4.7	0.4 ± 0.6	+31.0
0727	gi,re	DNA gyrase subunit A (GyrA)	5.9 ± 2.2	0.9 ± 0.0	+6.9
0235	m,cb,as	Phosphoglucomutase/phosphomannomutase family protein	5.7 ± 0.1	0.9 ± 0.1	+6.2
1610	rg	Putative bifuntional enzyme: tRNA methyltransferase; 2−C−methyl−D−erythritol 2,4−cyclodiphosphate synthase	5.0 ± 0.9	0.9 ± 0.1	+5.3
1090	gi,t	30S ribosomal protein S2 (Rps2,RpsB)	7.4 ± 0.2	1.7 ± 0.0	+4.3
1145	ukn	Hypothetical protein	5.2 ± 3.2	1.3 ± 0.6	+4.0
0670	ukn	Hypothetical protein	8.6 ± 4.2	2.2 ± 1.9	+4.0
0278	m,nu,py	Deoxycytidine triphosphate deaminase (Dcd)	8.1 ± 0.9	2.1 ± 0.7	+3.8
0827	ukn	Hypothetical protein	12.9 ± 2.3	3.4 ± 0.1	+3.8
0340	ukn	Hypothetical protein	7.4 ± 0.2	2.1 ± 0.7	+3.5
1171	m,a,gi,t	aminotransferase class−I (AspC)	7.6 ± 2.5	2.6 ± 0.1	+3.0
0402	m,l	Possible acyl carrier protein	5.0 ± 0.9	2.1 ± 0.5	+2.3
1852	m,l	3−oxoacyl−[acyl carrier protein] reductase (FabG)	5.9 ± 2.2	2.6 ± 0.1	+2.3
0144	ps	Photosystem I subunit VII (PsaC)	10.2 ± 4.1	4.7 ± 1.9	+2.2
0535	ps	Ferredoxin (PetF4)	12.7 ± 4.7	6.0 ± 0.1	+2.1
1936	abc,s	Putative sulfate transporter	5.2 ± 3.2	2.6 ± 2.5	+2.0
1091	gi	Elongation factor Ts (Tsf)	0.9 ± 1.3	10.2 ± 1.5	−11.0
1816	ukn	Hypothetical protein	0.7+1.0	6.8 ± 1.0	−9.4
0303	ps	Photosystem II manganese−stabilizing polypeptide (PsbO)	7.4 ± 0.2	35.7 ± 7.5	−4.8
1495	m,v,po,chl	Uroporphyrinogen decarboxylase (HemE)	1.7 ± 0.3	7.7 ± 2.6	−4.6
2139	gi,t	30S ribosomal protein S10 (Rps10, RpsJ)	1.2 ± 0.3	5.5 ± 1.7	−4.5
2081	gi,t	50S ribosomal protein L6 (Rpl6,RplF)	2.4 ± 0.7	10.2 ± 2.1	−4.3
0852	ukn	Hypothetical protein	1.7 ± 0.3	5.5 ± 0.5	−3.3
2069	gi,t	50S ribosomal protein L23 (Rpl23,RplW)	2.6 ± 1.6	8.5 ± 1.0	−3.3
2500	m,cb,tca, e,c	Aconitate hydratase (AcnB)	1.7 ± 0.3	5.1 ± 1.3	−3.1
2356	gi,t	Aspartyl/glutamyl−tRNA amidotransferase subunit B (GatB)	2.4 ± 0.7	7.3 ± 2.0	−3.0
1933	m,v,po,chl	δ-aminolevulinic acid dehydratase (HemB)	2.4 ± 0.7	7.2 ± 1.6	−3.0
1025	m,a	Putative anthranilate synthase component II (TrpD/G)	3.1 ± 1.8	9.0 ± 0.8	−2.9
2074	gi,t	50S ribosomal protein L16 (Rpl16,RplP)	2.4 ± 0.7	6.8 ± 2.2	−2.8
0045	u,m	Soluble hydrogenase small subunit (DHSS)	2.6 ± 1.6	7.2 ± 2.8	−2.8
1982	ps	Photosystem II chlorophyll−binding protein CP47 (PsbB)	4.8 ± 1.5	13.2 ± 2.	−2.8
0032	gi	Putative cyclophilin−type peptidyl−prolyl cis−trans isomerase	2.2 ± 3.1	6.0 ± 0.1	−2.7
0033	gi,t	Elongation factor P (Efp)	2.4 ± 0.7	6.4 ± 2.8	−2.7
0750	m,cb,g	Glucose−6−phosphate dehydrogenase (Zwf)	4.0 ± 0.4	10.7 ± 2.1	−2.6
0462	ei,si	Nitrogen regulatory protein P−II (GlnB)	2.6 ± 1.6	6.8 ± 1.0	−2.6
0819	m,a	Dihydrodipicolinate reductase (DapB)	2.7 ± 2.4	6.8 ± 1.0	−2.5
2067	gi,t	50S ribosomal protein L3 (Rpl3,RplC)	4.9 ± 5.5	12.0 ± 3.9	−2.5
2082	gi,t	50S ribosomal protein L18 (Rpl18,RplR)	3.1 ± 1.8	7.7 ± 0.2	−2.5
2442	abc,si	putative urea ABC transporter, urea binding protein (UrtA1)	24.1 ± 5.0	55.9 ± 4.5	−2.3
1815	abc,p	ABC transporter, substrate binding protein, phosphate (PstS)	4.0 ± 0.4	9.4 ± 1.0	−2.3
1694	gi,t	30S ribosomal protein S4 (Rps4,RpsD)	4.1 ± 4.4	9.4 ± 0.2	−2.3
2348	m,cb,e,gi	Enolase (Eno)	7.0 ± 4.5	5.7 ± 2.6	−2.3
1824	gi,t	50S ribosomal protein L19 (Rpl19)	6.6 ± 1.2	14.5 ± 0.4	−2.2
0687	m,nu,py	Putative thioredoxin reductase	2.4 ± 0.7	5.1 ± 1.3	−2.1
2246	ei	Two−component response regulator (RpaB)	5.7 ± 0.1	11.9 ± 2.1	−2.1
2137	gi,t	Elongation factor EF−2 (FusA)	12.1 ± 1.3	25.1 ± 1.2	−2.1
1835	ps	Photosystem I reaction center subunit III (PsaF)	3.3 ± 0.6	6.9 ± 2.6	−2.1
1718	c	Ribulose bisphosphate carboxylase, large chain (RbcL,CbbL)	38.2 ± 11.7	78.7 ± 9.4	−2.1
0405	m,nu,pu,a	Fumarate lyase: adenylosuccinate lyase (PurB)	3.1 ± 1.8	6.4 ± 0.8	−2.1
1617	gi,t	30S ribosomal protein S16 (Rps16,RpsP)	4.8 ± 1.5	9.8 ± 2.1	−2.1
2487	abc,ei	Putative cyanate ABC transporter	5.5 ± 2.5	11.1 ± 2.7	−2.0
0514	gi,f	Chaperonin (GroEL)	55.8 ± 15.5	112.0 ± 0.1	−2.0
0613	u,gi,t	DNA−directed RNA polymerase beta subunit (RpoB)	4.0 ± 0.4	8.1 ± 1.6	−2.0
2068	gi,t	50S ribosomal protein L4 (Rpl4,RplD)	5.6 ± 6.5	11.1 ± 1.5	−2.0
1073	m,e,n,a	Glutamine synthetase, glutamate−ammonia ligase (GlnA)	20.0 ± 4.5	39.6 ± 1.6	−2.0

*Units are spectral counts*.

*Arranged in highest to lowest fold change Zn-high PO_4_^3−^, than Zn-low PO_4_^3−^. +, fold greater than Zn-high PO_4_^3−^; −, fold less than Zn-high PO_4_^3−^; ukn, unknown; gi, genetic information processing; re, DNA replication and repair; m, metabolism; cb, carbohydrate metabolism; as, amino sugar metabolism; rg, regulatory function; t, translation; nu, nucleic acid metabolism; py, pyrimidine metabolism; a, amino acid metabolism; l, lipid metabolism; ps, photosynthesis; abc, ABC transporter; s, sulfur metabolism; v, vitamin metabolism; po, porphyrin metabolism; chl, chlorophyll metabolism; tca, citrate cycle; e, energy metabolism; c, carbon fixation; g, glutathione metabolism; ei, environmental information processing; si, signaling; p, phosphorus metabolism; f, protein folding; pu, purine biosynthesis; n, nitrogen metabolism*.

We also examined the proteome PO_4_^3−^ response in the presence and absence of Zn with the added interaction of Cd. 17 proteins were two-fold or more differentially abundant in the presence of Zn, 12 proteins with no added Zn (Supplementary Tables 1A,B). Nine proteins were more abundant in the Zn/low PO_4_^3−^/short-term Cd treatment, including phosphate stress proteins. Eight proteins were more abundant in the Zn/high PO_4_^3−^/short-term Cd treatment, including three related to the phycobilisomes and two ribosomal proteins. Six of the eight proteins more abundant in the no Zn/high PO_4_^3−^/short-term Cd treatment were involved in photosynthesis.

**Figure 8:**
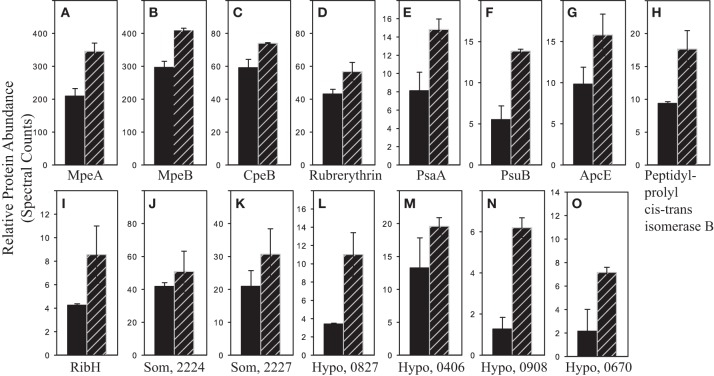
**Proteins more abundant with short-term cadmium addition**. Relative protein abundances of proteins two-fold or more greater in abundance and/or statistically different by Fisher's Exact Test in the no Zn/65 μM PO_4_^3−^/+ 4.4 pM Cd^2+^ (hatched bars) compared to the no Zn/65 μM PO_4_^3−^ treatments (solid bars). **(A)** SYNW2009 C-phycoerythrin class II, α chain, **(B)** SYNW2008 C-phycoerythrin class II, β chain, **(C)** SYNW2017 C-phycoerythrin class I, β chain, **(D)** SYNW2369 rubrerythrin, **(E)** SYNW2124 PSI P700 (PsaA), **(F)** SYNW3191 PSII extrinsic precursor (PsuB), **(G)** SYNW0486 anchor polypeptide L_CM_ (ApcE), **(H)** SYNW1264 peptidyl-prolyl cis-trans isomerase, **(I)** SYNW0082 riboflavin synthase subunit b (RibH), **(J)** SYNW2224 possible porin (Som, 2224), **(K)** SYNW2227 possible porin (Som, 2227), **(L)** SYNW0827 hypothetical protein (Hypo, 0827), **(M)** SYNW0406 hypothetical protein (Hypo, 0406), **(N)** SYNW0908 hypothetical protein (Hypo, 0908), and **(O)** SYNW0670 hypothetical protein (Hypo, 0670). Error bars are the standard deviation of duplicate injections.

**Figure 9:**
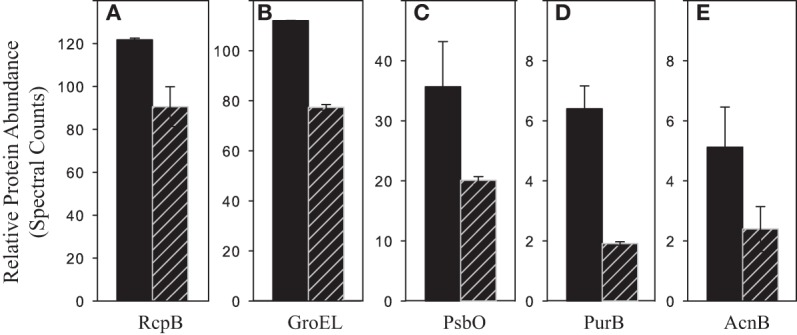
**Proteins more abundant without short-term cadmium addition**. Relative protein abundances of proteins more than two-fold less abundant and/or statistically different by Fisher's Exact Test in the no Zn/65 μM PO_4_^3−^/+ 4.4 pM Cd^2+^ (hatched bars) compared to the no Zn/65 μM PO_4_^3−^ treatments (solid bars). **(A)** SYNW2022 R-phycocyanin II, β chain (RpcB), **(B)** SYNW0514 chaperonin GroEL (GroEL), **(C)** SYNW0303 PSII Mn-stabilizing polypeptide (PsbO), **(D)** SYNW0405 fumarate lyase: adenylosuccinate lyase (PurB), and **(E)** SYNW2500 aconitate hydratase (AcnB). Error bars are the standard deviation of duplicate injections.

Cd-specific effects were discerned by examining pairwise protein comparisons (Figure 5). Cd effects were expected to be more pronounced with no added Zn. In the no Zn/high PO_4_^3−^/short-term Cd^2+^ compared to no Cd^2+^ added treatments, 10 proteins were two-fold or more differentially abundant (Table 3). Five proteins were more abundant in the no Zn/high PO_4_^3−^/short-term Cd^2+^ treatment including three unknown proteins and one involved in photosystem II (Figure 8; Table 3). Five proteins were more abundant in the no Zn/high PO_4_^3−^/no added Cd^2+^ treatment (Figure 9; Table 3). In addition, 10 proteins significantly different by Fisher's Exact Test are included in Figure 8 (five involved in photosynthesis) and 3 (two involved in photosynthesis) in Figure 9 (Supplementary Table 1C). The other three Zn and PO_4_^3−^ conditions for cadmium comparison showed some differences upon Cd addition. At high PO_4_^3−^, short-term Cd addition in the presence of Zn caused 4 proteins to be differentially abundant (Supplementary Table 1D). At low PO_4_^3−^ with no Zn, 32 proteins were differentially abundant, whereas with added Zn, only 7 (Supplementary Tables 1E,F).

**Table 3:** **Relative protein abundances between added (+ 4.4 pM Cd^2+^) and no added Cd treatments for proteins two-fold or greater differentially abundant (phosphate replete and scarce Zn for both)**.

**SYNW ID**	**KEGG Function**	**Protein**	**+4.4 pM Cd^2^+**	**No added Cd^2^+**	**Cd^2^+/no Cd^2^+ fold change**	**Fisher test *P*-value**
0908	ukn	Hypothetical protein	6.2 ± 0.5	1.3 ± 0.6	+4.9	95% (0.01)
0670	ukn	Hypothetical protein	7.2 ± 0.4	2.2 ± 1.9	+3.3	95% (0.0048)
0827	ukn	Hypothetical protein	11.0 ± 2.4	3.4 ± 0.1	+3.2	95% (0.0016)
2191	ps	Photosystem II complex extrinsic protein precursor (PsuB)	13.8 ± 0.2	5.5 ± 1.7	+2.5	95% (0.0016)
0082	m,v,r	Riboflavin synthase subunit beta (RibH)	8.6 ± 2.4	4.3 ± 0.1	+2.0	95% (0.047)
1118	m,cb	Glucose−1−phosphate adenylyltransferase (Agp, GlgC)	1.5 ± 0.7	5.5 ± 0.5	−3.6	95% (0.019)
0405	m,nu,pu,a	fumarate lyase: adenylosuccinate lyase (PurB)	1.9+0.1	6.4 ± 0.8	−3.4	95% (0.041)
2139	gi,t	30S ribosomal protein S10 (Rps10, RpsJ)	1.9 ± 0.1	5.5 ± 1.7	−2.9	0% (0.09)
1953	ukn,l	Putative glycerol kinase	2.4 ± 2.1	5.6 ± 2.0	−2.3	0% (0.15)
2500	m,cb,tca,e,c	Aconitate hydratase (AcnB)	2.4 ± 0.7	5.1 ± 1.3	−2.1	0% (0.21)

*Units are spectral counts*.

*Arranged in highest to lowest fold change 4.4 pM Cd^2^+ vs. control (no Zn and no added Cd). +, fold greater than control; −, fold less than control; ukn, unknown; ps, photosynthesis; m, metabolism; v, vitamin metabolism; r, riboflavin metabolism; cb, carbohydrate metabolism; nu, nucleic acid metabolism; pu, purine metabolism; a, amino acid metabolism; gi, genetic information processing; t, translation; l, lipid metabolism; tca, citrate cycle; e, energy metabolism; c, carbon fixation, in this case reductive glyoxylate cycle*.

Proteins with differential abundances with respect to Zn are listed in Supplementary Tables 1G–J. Among those listed are proteins involved in many cellular processes, ranging from photosynthesis to lipid metabolism. Notable were four proteins more abundant in the Zn/low PO_4_^3−^/short-term Cd^2+^ treatment compared to the no Zn/low PO_4_^3−^/short-term Cd^2+^, including SYNW0359 bacterial metallothionein and SYNW2391 putative alkaline phosphatase (Figure 7).

Comparing the proteomic response of the presence of either Cd or Zn at high PO_4_^3−^ queried if Cd could potentially “replace” Zn (Figure 2 - black/hatched to blue). In the no Zn/high PO_4_^3−^/short-term Cd^2+^ compared to Zn/high PO_4_^3−^ treatments, 8 proteins were two-fold or more differentially abundant (Supplementary Table 1K). Seven proteins were more abundant in the no Zn/high PO_4_^3^/short-term Cd^2+^, including four proteins involved in photosynthesis, a cell surface protein required for swimming motility (SwmA) and a possible outer membrane associated porin with unknown function. Aconitate hydratase, involved in carbohydrate metabolism, the TCA cycle, energy metabolism, and the reductive carboxylate cycle was more abundant in the Zn/high PO_4_^3−^ treatment.

## Discussion

We examined the interactive influences of PO_4_^3−^, Zn, and Cd on the proteome of *Synechococcus* WH8102. Three variables in the experimental design [long-term PO_4_^3−^ and Zn limitation and short-term Cd additions] and the dense nature of proteomic datasets yielded complex results as described in Results and Data Tables. Nevertheless, four main observations arose from these combined experiments. First, PO_4_^3−^ limitation had the largest effect on the proteome and showed numerous commonalities to a prior PO_4_^3−^ limitation transcriptome study (Tetu et al., 2009). Second, low Zn availability had an effect on the PO_4_^3−^ response, implying a critical role for this micronutrient in the PO_4_^3−^ response system. Third, metallothionein covaried with PO_4_^3−^ stress-associated proteins, implying a role in metal homeostasis and perhaps in supplying metals for metalloproteins such as alkaline phosphatase. And fourth, short-term Cd addition incubations were found to have the greatest influence on the proteome at low PO_4_^3−^ and Zn, compared to under both replete PO_4_^3−^ and Zn, implying an inability to confront Cd/Zn imbalance. A number of other intriguing details were observed during these experiments including different relative abundance levels of alkaline phosphatase isoforms and many hypothetical proteins identified. These findings will be expanded upon in the following sections.

### Phosphate and zinc influences on proteome

The long-term PO_4_^3−^ and Zn limitation matrix experiments allowed us to examine the influences of nutrient scarcity, both independently and synergistically. As described in Results, PO_4_^3−^ appeared to cause the largest difference in this Cd-Zn-PO_4_^3−^ interaction experiment (Figures 4, 5). The proteome and transcriptome showed similarity in responses (Figure 6; Tables 1,2). Tetu et al. (2009) identified 36 genes as having increased transcript expression under PO_4_^3−^ stress [PO_4_^3−^ limitation was observed at 5 μM PO_4_^3−^ with replete at 87 μM PO_4_^3−^ in their study]. We identified 13 of those genes as proteins in the two PO_4_^3−^ treatments with Zn (Figure 6). Six of these proteins were at least two-fold more abundant in the low PO_4_^3−^ treatment (Figure 6). This means that we identified as proteins 36% of the genes upregulated in the transcriptome (13 proteins of 36 transcripts) with 17% (6 proteins of 36 transcripts) also being comparable to strongly upregulated in the transcriptome (Figure 6). This coherence between the transcriptome and proteome under PO_4_^3−^ limiting conditions has also been observed in two eukaryotic phytoplankters, *Aureococcus anophagefferens* and *Thalassiosira pseudonana* (Wurch et al., 2011; Dyhrman et al., 2012), suggesting that adaptive responses to PO_4_^3−^ stress and proteome/transcriptome coordination are common among marine phytoplankton. Moreover, since observations of coordination between the transcriptome and proteome have thus far been relatively recent in the literature, this study contributes to the notion that the scalable cellular capability for PO_4_^3−^ stress contributes to the coordination we have observed thus far amongst these three distinct marine microbes. In particular, the alkaline phosphatase (PhoA, SYNW2391), a protein that requires a metal cofactor (identified as Zn in model organisms), and the phosphate binding protein (PstS, SYNW1019) were identified as more abundant in both the proteome and transcriptome under PO_4_^3−^ stress. In addition, among the 20 proteins with increased abundance under PO_4_^3−^ limitation were bacterial metallothionein (SmtA), a cell surface protein required for swimming mobility (SwmB), and three hypothetical proteins (SYNW0128, SYNW0160 and SYNW1661), suggesting that increased acquisition capability, motility, and the other proteins are also important to phosphate limitation. These observations may be related to the use of culture medium that closely resembles the metal micronutrient conditions of the open ocean.

Less overlap was observed between the transcriptome and the proteome for downregulated transcripts. Of the 23 transcripts observed strongly downregulated (greater than or equal to two-fold less) in the transcriptome study only 3 were identified as proteins, two ribosomal proteins and a conserved hypothetical protein. Of these three, only one was two-fold less in abundance (Table 1). One might expect less overlap in protein abundances from downregulated transcripts because as less protein is made detection becomes more difficult under the stringent identification parameters used here.

It is interesting to note that four of the mentioned PO_4_^3−^ acquisition genes, in addition to others, increased in expression in a microarray experiment when WH8102 was grown with *Vibrio parahaemolyticus*, a model heterotroph (Tai et al., 2009). This information combined with results from previous protein experiments in which these PO_4_^3−^ acquisition genes were more abundant with short-term Cd addition in WH8102 stationary phase cultures (Cox and Saito, unpublished data), suggests that the PO_4_^3−^ response may ultimately be triggered by many factors, including limitation experienced as a culture ages. The techniques used in this study could be applied to multi-organism experiments.

The response to a combination of Zn and PO_4_^3−^ scarcity was considerably different, particularly for proteins in high abundance under PO_4_^3−^ scarcity (Figure 5A; Table 2). None of the proteins were the same as the Tetu et al. (2009) transcriptome study. Most notable was the influence on two key PO_4_^3−^ acquisition proteins: the alkaline phosphatase and phosphate transporter described above (Figures 7B,C), which were only modestly affected by PO_4_^3−^ limitation under Zn scarcity. In addition, bacterial metallothionein did not increase in abundance with scarce PO_4_^3−^ (Figure 7A). Together these responses suggest a regulatory response to Zn that prevents synthesis of the metalloenzyme alkaline phosphatase when a necessary metal cofactor is absent. We should caveat that the metal atom center has not been demonstrated to be Zn for this alkaline phosphatase isoform, and other metals may also have functionality (or even be the “intended” metal), and that marine cyanobacteria including *Synechococcus* sp. WH8102, *S. bacillaris*, and *Prochlorococcus* MED4 have all been shown to have little to no Zn requirement (Sunda and Huntsman, 1995; Saito et al., 2002, 2003), although this has not been tested under conditions of organic PO_4_^3−^ utilization. In addition, our results suggest that the hypothetical protein SYNW1661 may be involved in the phosphate stress response in the presence of zinc (Table 1). Together, these observations suggest that Zn nutritional levels are connected to the PO_4_^3−^ response in this cyanobacterium.

Many proteins decreased in abundance in response to PO_4_^3−^ scarcity under low Zn conditions, such as a number of ribosomal proteins found in lower abundance that are likely related to the depressed growth rates (Table 2). A number of hypothetical proteins were also observed to increase in response to PO_4_^3−^ stress under Zn scarcity, including SYNW0380, 1145, 0670, 0827, and 0340 (Table 2). These proteins could be responsible for PO_4_^3−^ acquisition and utilization at scarce Zn and PO_4_^3−^, levels consistent with conditions encountered by cyanobacteria in the ocean. SYNW0380 could be directly involved in metal binding. It contains 239 total amino acid residues, 10 of these are cysteine and 14 histidine. There are two domains with the sequence -CXXC-.

### Response of alkaline phosphatase isoforms to low phosphate

Four genes in the genome of WH8102 are annotated as alkaline phosphatases, SYNW0120 putative alkaline phosphatase-like protein, SYNW0196 putative alkaline phosphatase, SYNW2390 putative alkaline phosphatase/5′ nucleotidase, and SNW2391 putative alkaline phosphatase (*phoA*). In addition, SYNW1799 is an alkaline phosphatase (*phoX*) (Kathuria and Martiny, 2011). Alkaline phosphatases vary in cellular location and associated metal ions. Two alkaline phosphatases purified from different strains of *Vibrio cholerae*, a γ-proteobacteria, acted on a variety of organic PO_4_^3−^ esters, but showed different levels of reactivation upon addition of Na^+^, K^+^, and Mg^2+^ ions (Roy et al., 1982). Some alkaline phosphatases (PhoA) are thought to be located in the periplasm and are activated by Zn and Mg, whereas other alkaline phosphatases (PhoX, PhoD) are activated by calcium ions (Ca^2+^) (Luo et al., 2009). A recent survey of the metagenomic databases concluded that *phoX* appeared to be more widespread in the ocean than *phoA* (Sebastian and Ammerman, 2009). There are also other types of alkaline phosphatases in cyanobacteria. The freshwater cyanobacterium *Synechococcus* 7942 contains a *phoV* in addition to *phoA* (Wagner et al., 1995). PhoV had broad substrate specificity for phosphomonoesters, required Zn^2+^ for activity and was inhibited by PO_4_^3−^, but was inhibited by Mn^2+^ (Wagner et al., 1995). Recent experimentation on PhoX (SYNW1799) overexpressed in *E. coli* have shown enhanced enzyme activity in the presence of Ca, leading the authors to conclude that bacterial lineages with the presence of *phoX* in the genome may not be subject to Zn-P colimitation (Kathuria and Martiny, 2011).

We detected SYNW2391 and SYNW1799, but not SYNW0120, SYNW2390 or SYN0196 as proteins in this experiment. SYNW2391 alkaline phosphatase (PhoA) is depicted in Figure 7, but SYNW1799 alkaline phosphatase (PhoX) was only detected by a few counts without significant abundance changes in our experimental matrix using our current detection capabilities, implying it is a relatively low abundance protein. This observation is contrary to what one might expect from a PhoX that does not presumably require Zn. Due to the high ratio of Ca/Zn in the ocean and in our medium, one would expect either low Zn or PO_4_^3−^ to result in the abundance of a Ca-alkaline phosphatase, particularly if the Ca-alkaline phosphatase has a lower specific activity than Zn-alkaline phosphatases. These protein results suggest that PhoX may not be as important as recently stated in the literature by metagenomic analysis by Sebastian and Ammerman (2009), assuming extrapolation from this physiological culture experiment to natural populations of cyanobacteria in the ocean, although further study would be required on this point.

### Metallothionein in *Synechococcus* WH8102

Metallothioneins are small, cysteine-rich, approximately 56 amino acid residue proteins involved in chelating metals such as Zn, Cd, copper (Cu), silver, mercury, and arsenic (Duncan et al., 2006). Their exact function is elusive but metallothioneins may function as (i) metal resistance proteins for detoxifying Zn, Cd, and Cu; (ii) reservoirs for the storage of excess Zn and/or Cu than can be mobilized under metal limiting conditions; (iii) metal chaperones that deliver Zn to Zn-dependent proteins; and/or (iv) antioxidants that scavenge oxygen radicals (Palmiter, 1998). They are known to bind, sequester, and buffer intracellular Zn in freshwater cyanobacteria (Robinson et al., 2001). Metallothionein relative protein abundances in this study were elevated with Zn added and interestingly this effect was accentuated by low PO_4_^3−^, suggesting a possible link to PO_4_^3−^ acquisition since alkaline phosphatase requires Zn (Figure 7). It seems likely that metallothionein could be acting as a metal reservoir supplying alkaline phosphatase with Zn. More quantitative analyses using a triple quadrupole mass spectrometer would be useful to constrain metallothionein change in WH8102. Ultimately, metallothionein may have developed as a relatively simple protein solution for cyanobacteria to cope with changing metal concentrations and increasing oxidation of the oceans over time, and may be important in the handling of Zn, Cd, and Cu in these organisms in the modern ocean.

### Influences of short-term Cd exposure

We also explored the influences of Cd addition on *Synechococcus* with a varying matrix of Zn and PO_4_^3−^ conditions. Previous studies noted the chemical correlation of Cd with PO_4_^3−^ in the ocean (Boyle et al., 1976; Boyle, 1988; Elderfield and Rickaby, 2000; Hendry et al., 2008), Cd replacement of Zn in the enzyme carbonic anhydrase (Lee et al., 1995; Lane et al., 2005; Xu et al., 2008), and have hypothesized that Cd replaces Zn in alkaline phosphatase (Morel et al., 2003). In this study, we observed a more pronounced Cd response during Zn and PO_4_^3−^ scarcity compared to replete conditions of each, suggesting that the sensitivity of natural populations to representative concentrations of Cd inputs may be greater than shown from culture studies performed with higher than ambient concentrations. We briefly discuss six proteomic responses in the following paragraphs: (1) Cd sensitivities at low nutrient concentrations, (2) Zn sensitivities at low PO_4_^3−^, (3) a buffering effect of Zn for Cd and effects on (4) photosynthetic (5) carbohydrate metabolism and (6) unknown function proteins. We finish by discussing the curious physiological response.

The WH8102 proteome was Cd-sensitive at lower nutrient concentrations. At low PO_4_^3−^, Cd had a greater effect on the proteome, based on the greater overall number of differentially abundant proteins (Figure 5B). Under scarce Zn conditions, Cd additions resulted in 32 proteins differentially abundant at low PO_4_^3−^ (Figure 5B, Supplementary Table 1E), compared to only 10 proteins differentially abundant in total at high PO_4_^3−^ (Figure 5B; Table 3). Cd addition at low PO_4_^3−^ resulted in three hypothetical proteins of unknown function becoming less abundant, suggesting a unique response to scarce nutrients (Table 3). These proteins could be important to nutrient acquisition in natural populations, warranting further scrutiny. In addition, this organism may be more vulnerable to Cd with scarce Zn because only four proteins were more abundant in the no Zn/low PO_4_^3−^/short-term Cd (Figure 5A, Supplementary Table 1B), including SwmB and PstS. Because these two proteins were not differentially abundant at no Zn/low PO_4_^3−^, perhaps short-term Cd addition stimulated the presence of these proteins (Table 2).

Short-term Cd exposure also showed an influence when varying Zn abundances particularly in the low PO_4_^3−^ treatments (Supplementary Table 1). With Cd exposure under low Zn, a component of the ABC phosphate transporter (SYNW1815, provisional PstS) and four other proteins were more abundant (Figure 5C, Supplementary Table 1J), whereas added Zn resulted in four more abundant proteins including bacterial metallothionein, putative alkaline phosphatase, and probable glutathione reductase (NADH) (Figures 5C, 7, Supplementary Table 1J). Glutathione may be involved in intracellular Cd binding. As mentioned above, greater metallothionein and alkaline phosphatase abundances with added Zn are consistent with Zn involvement in these proteins, either by being bound or in the active site.

The Cd sensitivity described above was largely ameliorated with added Zn (Figure 5A; Table 1, Supplementary Table 1A). For example, 5 of the 9 proteins more abundant at Zn/low PO_4_^3−^/short-term Cd relative to Zn/high PO_4_^3−^/short-term Cd were also differentially abundant at Zn/low PO_4_^3−^ without Cd addition (Figure 5A; Table 1, Supplementary Table 1A). Four of these five proteins were also expressed as transcripts in the microarray experiment and are PO_4_^3−^ stress-related (Figure 5A; Table 1, Supplementary Table 1A). Bacterial metallothionein is the fifth protein, only found in the replete Zn without Cd addition (Figure 7). The presence of these proteins in Zn treatments suggests that the main proteins known to be involved in the PO_4_^3−^ response were more responsive to the presence of Zn than Cd.

Heavy metal interference in photosynthesis has been previously observed in plant systems (Sujak, 2005). As well as phycobilisome proteins observed during PO_4_^3−^ scarcity, six of the eight proteins more abundant in the scarce Zn short-term Cd^2+^ high PO_4_^3−^ treatment are involved in photosynthesis (two phycobilisome, three Photosystem II and one Photosystem I proteins) (Figure 5A), suggesting Cd interference in photosynthesis (Figure 5A, Supplementary Table 1A). These protein responses are consistent with the higher short-term growth rates after Cd addition, and Cd may have stimulated short-term carbon fixation at low PO_4_^3−^ (Figure 3, see next section). As with high PO_4_^3−^, differentially abundant proteins with Cd addition decreased with added Zn at low PO_4_^3−^ (Figure 5B, Supplementary Table 1G). Cd may have stimulated carbon fixation because δ-aminolevulinic acid dehydratase, an enzyme in the chlorophyll biosynthesis pathway, and putative carboxysome structural peptide (CsoS2), involved in carbon fixation, were more abundant. Yet, Cd addition may have also had negative metabolic impacts: the no added Cd treatment had five proteins differentially more abundant compared to with Cd addition, including a hypothetical protein and a protein involved in each of lipid, purine, carbohydrate, and amino acid metabolism (Supplementary Table 1G).

Short-term Cd exposure appeared to affect carbohydrate metabolism. Changes in genes and proteins associated with carbohydrate flux under oxidative and Cd stress has been observed in eukaryotic organisms (Godon et al., 1998; Ralser et al., 2007; Guo et al., 2012). In this study, Cd addition with scarce Zn and high PO_4_^3−^ caused five proteins to be significantly less abundant, including two involved in carbohydrate metabolism, two involved in photosynthesis and one in protein folding (Figures 5B, 7; Table 3, Supplementary Table 1C). Again, Cd in the absence of Zn may negatively affect the photosynthetic apparatus and additionally, carbohydrate production.

Three proteins of unknown function (SYNW0908, 0670 and 0827) became more abundant with Cd addition under scarce Zn and replete PO_4_^3−^ conditions (Figures 5B, 8; Table 3). An additional protein of unknown function (SYNW0406) was determined statistically different by Fisher's Exact Test (Figure 8; Supplementary Table 1C). These hypothetical proteins might be involved in Cd handling with scarce Zn or part of the general Cd response, because they were not differentially abundant with added Zn. Two of these proteins (SYNW0670 and 0827) are also more abundant with scarce Zn and PO_4_^3−^ stress. Five of the 10 additional proteins significantly different by Fisher's Exact Test in these two treatments are involved in photosynthesis further supporting Cd interference in the photosynthetic process (Figure 8; Supplementary Table 1C).

### A curious short-term physiological response to Cd addition at low PO_4_^3−^ and added Zn

Instantaneous (24 h) growth rates and higher cell abundances in the Zn/low PO_4_^3−^/short-term Cd addition imply that Cd may have been used as a nutrient (Figures 3F,G). This could result from direct Cd usage as a nutrient or more likely by release of a nutritive intracellular Zn pool due to Cd exposure; as discussed above metallothionein is one possible “Zn buffer” (Frausto da Silva and Williams, 1991) and in mammals upon Cd and Cu loading, metallothionein releases Zn (Zhang et al., 2003). The “nutritive” Cd effect was not observed in any other treatments, although all combinations of Zn and PO_4_^3−^ showed slight growth rates increases with short-term Cd addition and the Zn/low PO_4_^3−^ combination showed a slight increase in final cell abundances with short-term Cd addition. Only the Zn/low PO_4_^3−^ treatment showed a large difference in both. Instantaneous growth rates in the Zn treatments at both PO_4_^3−^ levels during the last 24 h increased by factors of ~2 and 1.7 with short-term Cd addition relative to no added Cd (Figure 3F). In contrast, hardly an increase in instantaneous growth rates was observed in the no Zn treatments, both low and high PO_4_^3−^ with the Cd addition relative to no Cd added (Figure 3F).

The low dosage Cd stimulation we observed may be a hormetic effect and the mechanism, albeit unknown, could be in the interaction with Zn. A hormetic response is defined as low dosage stimulation with higher dosage toxicity (Calabrese, 2005). Cd responses at varying concentrations would be required to observe a full hormetic curve, as has been documented in mammalian cellular systems (Misra et al., 2002, 2003; Mantha and Jumarie, 2010). Although the descriptor hormetic was not used, low Cd concentrations stimulated the growth of *Chlorella*, a photosynthetic eukaryotic organism, and inhibited growth at higher concentrations (Vallee and Ulmer, 1972). Alternative to Zn displacement by Cd, Cd could directly have a nutritive or regulatory effect inducing cell division, although the latter effect has only been observed in eukaryotic systems to date (Misra et al., 2002, 2003; Sobkowiak and Deckert, 2003). Non-redundant pBLAST searches of mitotic cyclin b1-type and p38 mitogen activated protein kinase [from eukaryotic systems studied by Misra et al. (2002) and Sobkowiak and Deckert (2003)] yielded no hits against *Synechococcus* sp. WH8102 (Altschul et al., 1997), suggesting this microbe's Cd response is not modulated by these systems as observed elsewhere. Using this data set, we cannot distinguish between nutritive effects of Cd caused by intracellular Zn release upon Cd exposure or because of Cd alone.

## Conclusions

In conclusion, the physiologic response of *Synechococcus* WH8102 to short-term Cd^2+^ addition under four varying Zn and PO_4_^3−^ treatments [Zn/high PO_4_^3−^, no Zn/low PO_4_^3−^, no Zn/high PO_4_^3−^, and no Zn/low PO_4_^3−^] revealed during the last 24 h of the experiment relative to the high PO_4_^3−^ conditions: i) increased growth rates under low PO_4_^3−^ conditions and ii) even greater increased growth rates with Cd addition under low PO_4_^3−^ and Zn conditions. The proteomic response revealed differential abundances of PO_4_^3−^ stress proteins and differential protein abundances with chronic Zn and Cd addition. Considering the proteomic data, it appears that Zn nutrition is an important component of the known PO_4_^3−^ response in this organism because of the difference in response to PO_4_^3−^ with and without Zn. These findings are consistent with the ideas that Zn is beneficial for the functioning of alkaline phosphatase and other proteins involved in PO_4_^3−^ acquisition, and at environmentally relevant PO_4_^3−^ concentrations the presence of Zn and Cd make a difference in the physiology and proteome of cells, perhaps by influencing regulation. Greater abundances of hypothetical proteins in some treatments relative to others suggest these proteins may be involved in phosphate, cadmium and zinc stress or combinations thereof. Bacterial metallothionein appears to be regulated with alkaline phosphatase, suggesting a Zn-handling mechanism in which alkaline phosphatase is supplied with Zn by metallothionein. In addition to proteins of unknown function, Cd affected photosynthetic and carbohydrate metabolism proteins, and appeared to have the greatest overall effect on the proteome at low PO_4_^3−^ and Zn.

Comparison of proteomic data to literature transcriptome analyses shows a similar response of many important phosphate stress related proteins [putative alkaline phosphatase, periplasmic ABC phosphate binding protein (PstS), motility-related proteins (SwmA and SwmB), and possible porin)] but also shows other proteins that did not respond in the microarray study, such as bacterial metallothionein (SmtA), as well as proteins that did respond in the microarray study and not this one, like thioredoxin peroxidase. These data suggest that there is a fair amount of consistency between the transcriptome and proteome under phosphate stress. Taken together with the fact that the treatments without Zn showed a different proteomic reaction to phosphate stress, the presence of Zn appears important to the phosphorus metabolism of this open ocean cyanobacterium.

### Conflict of interest statement

The authors declare that the research was conducted in the absence of any commercial or financial relationships that could be construed as a potential conflict of interest.
